# Crystal Plasticity Finite Element Study on Orientation Evolution and Deformation Inhomogeneity of Island Grain During the Ultra-Thin Strips Rolling of Grain Oriented Electrical Steel

**DOI:** 10.3390/ma17246276

**Published:** 2024-12-22

**Authors:** Huanzhu Wang, Ping Yang, Qingge Xie, Xinfu Gu

**Affiliations:** 1 School of Materials Science and Engineering, University of Science and Technology Beijing, Beijing 100083, China; 2 Collaborative Innovation Center of Steel Technology, University of Science and Technology Beijing, Beijing 100083, China

**Keywords:** grain oriented electrical steel, ultra-thin strips, island grain, heterogeneity

## Abstract

The presence of island grains in the initial finished sheets of grain-oriented electrical steel is inevitable in the preparation of ultra-thin strips. Owing to their distinctive shape and size effects, their deformation behavior during rolling differs from that of grain-oriented electrical steels of conventional thickness. This study focuses on the orientation evolution and deformation heterogeneity of island grains during rolling. Four types of island grains with orientations of {210}<001>, {110}<112>, {114}<481>, and {100}<021> were selected and modeled within the Goss-oriented matrix using full-field crystal plasticity finite element (CPFEM) simulation under plane strain compression. The results are then compared with corresponding experimental measurements. The results reveal that orientation rotation and grain fragmentation vary among the island grains of different orientations, with the first two orientations exhibiting more significant deformation heterogeneity compared to the latter two. Additionally, the orientations of the island grains significantly affect the distribution of residual Goss orientations within the surrounding matrix. Pancake-like island grains exhibit a higher degree of orientation scatter and greater deformation heterogeneity in the central layer compared to their spherical counterparts. The initial {210}<001> island grains can form a cube orientation, which can be optimized by subsequent process control to enhance magnetic properties.

## 1. Introduction

Island grains, which are small grains less than 2 mm in size located within or at the boundaries of centimeter-scale Goss grains, are inevitably present in the finished sheets of grain-oriented electrical steel. Even in high-magnetic-induction grain-oriented electrical steel sheets with a magnetic induction value of B_8_ = 1.91 T, there are approximately 20 island grains per cm^2^ [1]. These grains could be secondary grain orientations, such as {210}<001>, {110}<112>, or {110}<227> orientations, or the matrix orientations before secondary recrystallization, such as near {114}<481> and {100}<021> orientations. Although the percentage of island grains is small, their presence reduces the magnetic properties of the finished sheets. Industrial production improves magnetic properties by reducing island grains. To achieve excellence, it is necessary to determine the causes of island grains and methods for their elimination or reduction, which have been extensively studied [2,3,4]. An ultra-thin strip of oriented silicon steel is prepared by the continuous casting, rolling, and annealing of industrial oriented steel products. Experimental methods are usually used to study the effects of initial texture, grain size, reduction, and annealing temperature on annealing texture and microstructure, without paying attention to the influence of island grains in the initial material. Moreover, if high-magnetic-induction grain-oriented electrical steels are used as materials for the subsequent production of ultra-thin strips (the thickness is generally 0.10 mm or less), the deformation and recrystallization behavior of island grains differ from those of the matrix Goss grains. The interaction between island grains of different sizes, quantities, or orientations, and the surrounding coarse Goss-oriented grains, differs during the cold rolling process. This variation leads to distinct regions of deformation inhomogeneity within the island grains themselves and in the Goss matrix near their interfaces, and the influence on subsequent recrystallization nucleation, texture evolution, and magnetic properties of the ultra-thin strip is also affected differently. Therefore, studying the orientation evolution of island grains is of practical value for controlling or optimizing the texture of ultra-thin strips. Currently, while there are many studies on the deformation and recrystallization process of ultra-thin strips, they are mostly focused on the deformation and recrystallization behavior of the Goss grains, with a lack of attention and theoretical research on island grains with different orientations [5,6,7,8,9,10,11]. In our previous quasi-in-situ detection [12], after cold rolling and recrystallization, these island grains were shown to produce unfavorable orientations, such as near {113}<361> orientation or {110}<112> Brass orientation, as well as favorable {100} orientation or even cube-oriented grain clusters. By simulation, these experimental characteristics can be partially or fully revealed, and the quantitative evolution of their organization and texture can be observed. In addition, we also focus on the deformation processes of two island crystals, {114}<481> and {100}<021>, which have not been studied in ultra-thin bands by previous researchers.

Simulating texture development and its impact on subsequent deformation requires material models that are capable of treating the distribution of crystal orientations and its evolution. In the full constrained Taylor model [13], the visco-plastic self-consistent model (VPSC) [14,15], and the elasto-plastic self-consistent (EPSC) model [16], the microstructure is represented in a statistical way, where each grain is considered to be homogeneous, thus disregarding realistic microstructural in-grain morphologies and specific local grain interactions. Only spatially averaged quantities can be obtained instead of the spatially resolved solutions. In the Grain Interaction (GIA) [17,18], advanced LAMEL (ALAMEL) [19,20,21], and Relaxed Grain Cluster (RGC) [22,23,24] models, as well as in Relaxed Constraints Taylor-type [25,26] models, the degrees of freedom are restricted to a small number of regions with homogeneous strain inside each zone. The mean field crystal plasticity simulation can be used as a material point simulator, and VPSC and ESPC can also be combined with finite element software to form mean field CPFEM [27,28,29]. The mean field crystal plasticity models can predict the macroscopic mechanical behavior (stress–strain curve) and statistical texture evolution of polycrystals well, but they struggle to predict the local, grain-scale microscopic mechanical behavior. Full-field homogenization refers to the spatially resolved solution of a representative volume element (RVE) by means of FEM [30,31,32] or spectral methods using a fast Fourier transform approach (FFT) [33,34,35,36]. Such a full-field homogenization approach can reach high precision in microstructure property simulations when using adequate crystal plasticity constitutive models and realistic microstructural morphologies.

Although the sizes of the island grains are small, they are much larger than the thickness of the sheets, so the cold rolling deformation behavior is similar to that of a two-dimensional, pancake-like island grain surrounded by large-sized Goss matrix grains (but the upper and lower surfaces are not affected by the matrix), which may be different from the deformation behavior of ordinary-thickness sheets with the same orientation grains. Based on previous experimental inspections [1,12], plane strain compression simulations are performed on island grains with four kinds of typical island grain orientations—{210}<001>, {110}<112>, {114}<481> and {100}<021>—using a full-field CPFEM. Two-dimensional cylindrical and three-dimensional spherical simulation blocks with {210}<001> and {110}<112> orientations are included. These simulations capture the inhomogeneities in deformation, not only across different grains, but also within each grain. In addition to examining the orientation change patterns under different reductions, special attention is paid to the characteristics of deformation inhomogeneity within the island grains and the surrounding Goss matrix, and the results are compared with actual measured data, with the aim of quantitatively describing the evolution of deformation texture and microstructure.

## 2. Materials and Methods

The experimental rolling involved in this study was introduced in the authors’ previous papers [1,6,8,12], which respectively investigated the influences of different reductions, annealing temperatures and initial Goss texture intensities, and various types of island grains, on the orientation evolution during the rolling and annealing of ultra-thin grain-oriented electrical steels. These aspects will not be reiterated here.

The full-field crystal plasticity model (CPFEM) employs finite element algorithms to solve partial differential equations under given boundary conditions, discretizing the grains through three-dimensional elements (meshes). The model can provide microstructural information after deformation, including grain size, morphology, grain topological relationships, grain boundaries and multiphase distributions. The employed full-field CPFEM follows the approach given in Ref. [37]. The deformation gradient tensor is written using a multiplicative decomposition, as shown in Equation (1),

(1) $$F=F*F^{p}$$

where $F^{p}$ represents the plastic deformation gradient tensor attributed to shearing along crystallographic slip planes, and F* is the elastic deformation gradient tensor, which includes lattice rotation and stretching. The plastic deformation by slip is assumed to occur at a constant volume, with the orientation of the lattice remaining unchanged. Re-expressing Equation (1) in a rate form leads to the following expression for the velocity gradient tensor L, as depicted in Equation (2),

(2) $$L=\dot{F}F^{-1}=\dot{F^{e}}{F^{e}}^{-1}+F^{e}\dot{F^{p}}F^{p-1}{F^{e}}^{-1}=L^{e}+L^{p}$$

where $L^{e}$ is the elastic velocity gradient tensor. $L^{p}$ is the plastic velocity gradient tensor, as detailed in Equation (3),

(3) $$L^{p}=\dot{F^{p}}{F^{p}}^{-1}=\sum_{\alpha }^{N}{\dot{\gamma }}^{\alpha }P^{\alpha }=\sum_{\alpha }^{N}{\dot{\gamma }}^{\alpha }m^{\alpha }{⊗n}^{\alpha }$$

where ${\dot{\gamma }}^{\alpha }$ is the shearing rate of the α slip system, *P*^α^ is the Schmidt tensor, and *m*^α^ and *n*^α^ are the unit vectors along the sliding direction and the normal to the slip plane of the α slip system, respectively.

The plastic deformation is modeled using a viscoplastic constitutive law to relate the shearing rate of each slip system ${\dot{\gamma }}^{\alpha }$ to its resolved shear stress $\tau^{\alpha }$. A rate-dependent model avoids the challenge of potential non-uniqueness in selecting the active slip systems [38,39]. The shearing rate is given by Equation (4),

(4) $${\dot{\gamma }}^{\alpha }=\dot{\gamma_{0}}{\left|\frac{\tau^{\alpha }}{{\hat{\tau }}^{\alpha }}\right|}^{\frac{1}{m}}sign\left(\tau^{\alpha }\right)$$

where $\dot{\gamma_{0}}$ is the reference shear rate, m is the rate sensitivity coefficient, and ${\hat{\tau }}^{\alpha }$ is the slip resistance of the α slip system, i.e., CRSS.

The shear stress is a component along the slip direction, which is connected by the Schmidt tensor and the Cauchy stress T, as shown in Equation (5),

(5) $$\tau^{\alpha }=(Tm^{\alpha })\cdot n^{\alpha }=({F^{e}}^{T}F^{e}T^{e})\cdot (m^{\alpha }⊗n^{\alpha })=({F^{e}}^{T}F^{e}T^{e})\cdot P^{\alpha }$$

where $T^{e}$ is the second Piola Kirchhoff stress tensor.

A modified Voce hardening law was used, as given in Equation (6),

(6) $$\dot{\tau^{\alpha }}=H_{0}(\frac{\tau_{s}-\tau^{\alpha }}{\tau_{s}-\tau_{0}})\Gamma$$

where *H*_0_ is proportional to the initial hardening rate, *τ*_0_ is the initial value of the critical resolved shear stress (CRSS) for initial yielding, and *τ_s_* is the saturation value of CRSS. Γ is the sum of the slip rates of all slip systems, as given by Equation (7),

(7) $$\Gamma =\sum_{\alpha =1}^{N}\left|\left.{\dot{\gamma }}^{\alpha }\right|\right.$$

where ${\dot{\gamma }}^{\alpha }$ is the shear rate on each slip system. The saturation hardening value *τ_s_* based on the current state of the slip system is given by Equation (8),

(8) $$\tau_{s}=\tau_{0}{\left(\frac{\Gamma }{{\dot{\gamma }}_{s}}\right)}^{n}$$

where *τ*_0_, ${\dot{\gamma }}_{s}$, and *n* are material parameters. In the simulation, the number of slip systems is set as 24, which includes {110}<111> and {112}<111> slip systems.

According to Hooke’s law, the constitutive equation of elastic deformation behavior for metal materials is shown in Equation (9), where C is a fourth-order elastic tensor, which can be represented by the elastic deformation gradient tensor $F^{e}$ and unit vector *I*.

(9) $$T^{e}=\frac{C}{2}\left({F^{e}}^{T}F^{e}-I\right){F^{e}}^{T}$$

Sarma et al. [40,41] proposed an implicit algorithm for calculating the elastic deformation gradient tensor and introduced it into finite element simulation. Upon obtaining the elastic deformation gradient tensor, the lattice rotation and stretching can be obtained through polar decomposition, as shown in Equation (10),

(10) $$F^{e}=R^{e}U^{e}$$

For BCC-structured electrical steels, four different orientations of island grains were embedded within Goss grains. The rolling direction (RD) used free boundary conditions, while the transverse direction (TD) used fixed boundary conditions and normal direction (ND) with plane strain compression for simulation. The texture orientation information is obtained using the Inverse Pole Figure parallel to the Z-axis (IPF-Z), where the RD corresponds to the X-axis, and the TD and ND correspond to the Y-axis and Z-axis, respectively. Each model is set with a 50 × 50 × 50 array of 8-node cubic elements. In the simulation, 24 slip systems are considered, including only the {110}<111> and {112}<111> slip systems, as the {123}<111> slip system in body-centered cubic crystals can be achieved by their superposition, with the ratio τ{110}<111>/τ{112}<111> set to 1. For the grain-oriented electrical steels, there are three stiffness coefficients—C_11_, C_12_, and C_44_ [42]. The relevant principles can be found in References [43,44]. Xie et al. [18,45] explored in detail the law of the hardening process of plastic, and conducted tests to provide a good prediction of the full-field microstructure evolution and crystallographic texture evolution of polycrystalline deformation. The CPFEM parameters for the four typical island grains microstructures are given in Table 1 under plane strain compression to approximate the rolling tests [42,45,46].

## 3. Results

### 3.1. CPFEM Microstructure and Orientations of {210}<001> Island Grain

**Figure 1:**
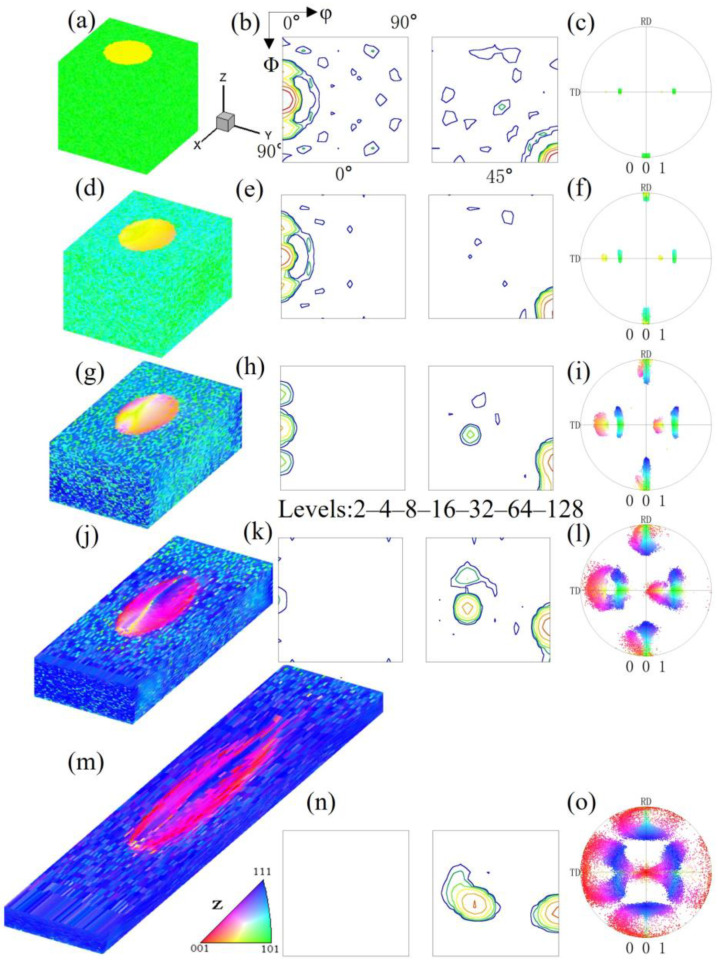
IPF-Z maps, ODF section and (001) pole figures of pancake-like {210}<001> island grain embedded in the Goss matrix with different reductions: (**a**–**c**) 0%, (**d**–**f**) 20%, (**g**–**i**) 40%, (**j**–**l**) 60%, (**m**–**o**) 80%.

Figure 1 shows the simulation data of the microstructure and orientation changes of pancake-like {210}<001> island grains embedded in the Goss matrix under various reductions. Figure 1a–c show the initial state of the {210}<001> island grains. Due to the small volume fraction of the island grains, their intensities in the calculated Orientation Distribution Function (ODF) and pole figures are lower than that of the Goss texture. After a 20% reduction, as shown in Figure 1d, the orientation within the island grains undergoes uneven rotation, with the Goss orientation slowly rotating towards {111}<112> orientation around the TD axis, changing from green to sky blue, while the green regions of residual Goss orientation are evenly distributed within the matrix. After 40% reduction, as shown in Figure 1g, the orientation within the {210}<001>-oriented island grains changes significantly. The Goss matrix has now rotated to the {111}<112> orientation, and two variants have emerged, but the matrix still contains evenly distributed residual Goss-oriented regions. The head and tail of the sample are less constrained, making them more conducive to turning {111}<112> orientations. Under 60% reduction, as shown in Figure 1j, the residual part of the island grains adopts near-cube orientation (orange), and most regions rotate to the {113}<361> orientation (pink). A part of the Goss-oriented matrix becomes two symmetrical near {111}<112> variant orientations, as shown in Figure 1l. Initial {210}<001> orientation regions and near {111}<112> orientation bands regions (blue) are distributed along the X-direction. At 80% reduction, as shown in Figure 1m, the {210}<001> island grains exhibit significant heterogeneity, with small regions of {100}<021> and residual {210}<001> orientation. The {113}<361> orientation gradually rotates towards {111}<110> orientation. There are blue {111}<112>-oriented regions near the grain boundaries and the top and bottom surfaces. However, the residual Goss regions no longer exist. Due to the low symmetry of the {210}<001> orientation in the orthogonal coordinate system, only one near-{113}<361> variant is formed under plane strain compression.

**Figure 2:**
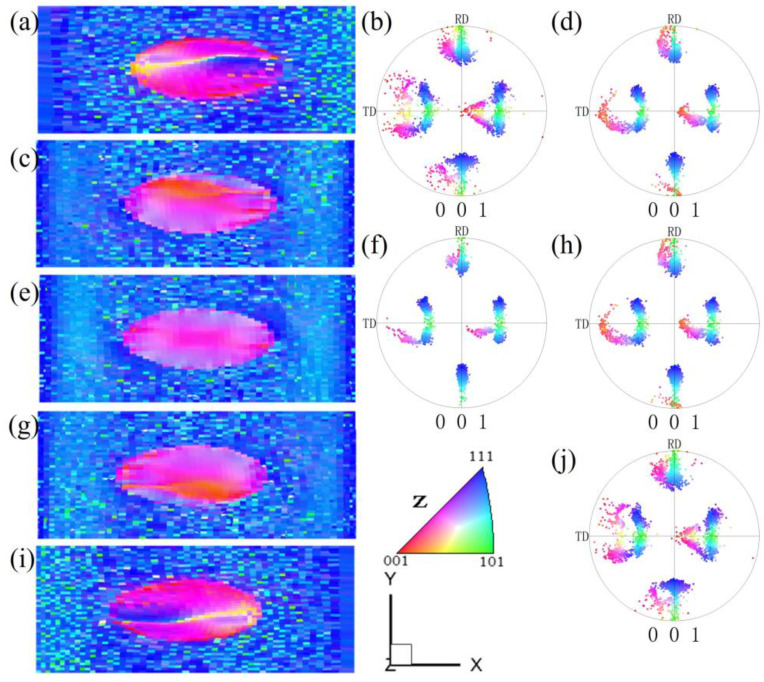
IPF-Z maps and (001) pole figures of the XY section with pancake-like {210}<001> island grains at 60% reduction: (**a**,**b**) z = 0, (**c**,**d**) z = 0.25, (**e**,**f**) z = 0.5, (**g**,**h**) z = 0.75, (**i**,**j**) z = 1.

Figure 2 shows the microstructure and orientation of pancake-like {210}<001> island grains at 60% reduction. Significant orientation changes in the {210}<001> island grain are observed, with bands parallel to the X direction, as shown in Figure 2a,c,e,g,i. The matrix Goss orientations rotate towards the {111}<112> orientations. In the upper and lower surfaces of the XY cross-section, as depicted in Figure 2a,i, the {210}<001> orientation rotates towards the purple {113}<361> and {114}<481> orientations, with residual yellow band-like {210}<001> orientations and a small amount of blue {111}<112> orientation. In Figure 2g,c, at the 0.25 and 0.75 layers of the XY cross-section, part of the {210}<001> island grain also rotates the orange {410}<001> orientation. In the central layer of the XY cross-section, as shown in Figure 2e, the degree of internal orientation inhomogeneity within the {210}<001> island grain decreases, with all rotating towards the {113}<361> and {114}<481> orientations.

**Figure 3:**
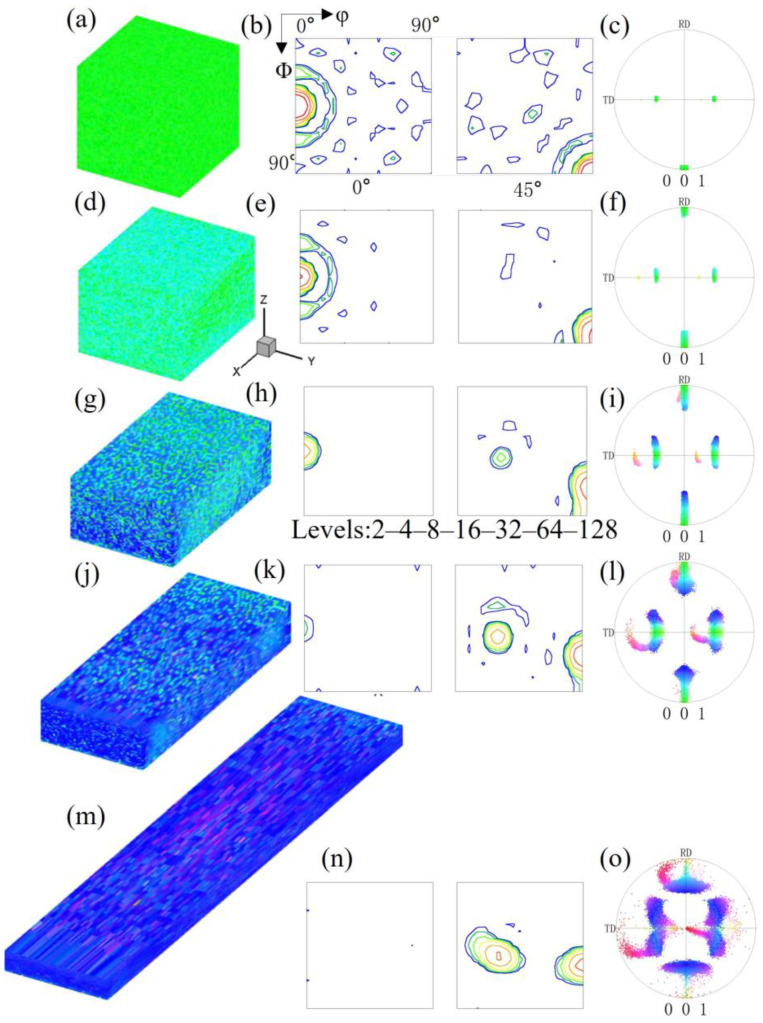
IPF-Z maps, ODF section and (001) pole figures of the spherical {210}<001> island grains embedded in the Goss matrix with different reductions: (**a**–**c**) 0%, (**d**–**f**) 20%, (**g**–**i**) 40%, (**j**–**l**) 60%, (**m**–**o**) 80%.

Pancake-like island grains exist with the top and bottom surfaces not being constrained by the matrix. To investigate the deformation characteristics of island grains of different shapes, we simulated the deformation process of spherical island grains, that is, the microstructure and orientation evolution of spherical island grains completely surrounded by the Goss matrix. Since the island grains cannot be observed in the overall microstructure, Figure 3 shows the overall microstructure and orientation of the spherical {210}<001> island grain under different reductions. It can be seen that, except near the grain boundaries, the overall orientation rotation manner is basically consistent with that of the pancake-like {210}<001> island grain simulation block; that is, the Goss orientation rotates towards the {111}<112> orientation, and the spherical {210}<001> orientation rotates towards the {113}<361> orientation. However, the spherical island grain simulation block has a lower degree of orientation rotation scatter, and only one near-{113}<361> variant is formed at 40% reduction. The inhomogeneous deformation distribution near the grain boundaries is quite different from that of the pancake-like {210}<001> island grain simulation block, where the near 25° cube orientation is formed at 80% reduction.

**Figure 4:**
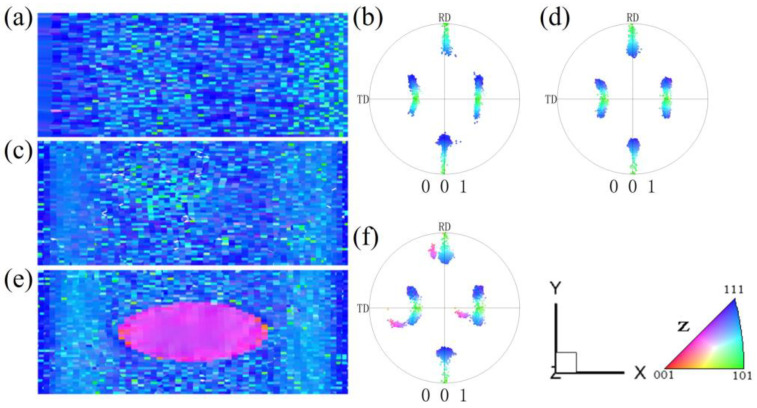
IPF-Z maps and (001) pole figures of the XY section with the spherical {210}<001> island grains at 60% reduction: (**a**,**b**) z = 0, (**c**,**d**) z = 0.25, (**e**,**f**) z = 0.5.

Figure 4 presents the XY cross-section microstructure and orientations of the spherical {210}<001> island grain embedded in a Goss matrix under 60% reduction. The information for the {210}<001> island grain is only extracted in the XY central cross-section, where the internal orientations of the island grain exhibit a low degree of deformation inhomogeneity, with almost all rotating towards the {113}<361> orientations.

**Figure 5:**
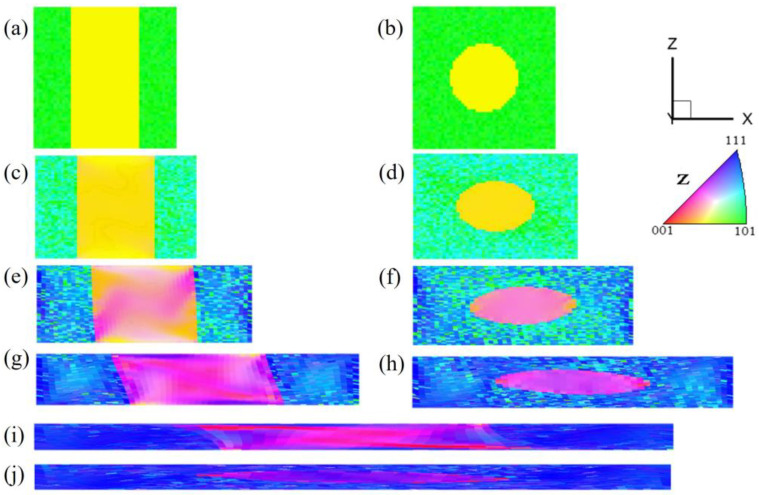
IPF-Z maps of XZ center section of pancake-like and spherical {210}<001> island grains with different reductions: (**a**,**b**) 0%, (**c**,**d**) 20%, (**e**,**f**) 40%, (**g**,**h**) 60%, (**i**,**j**) 80%.

**Figure 6:**
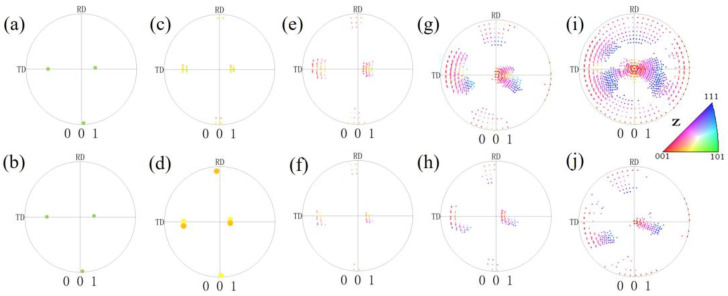
(001) pole figures of the pancake-like and spherical {210}<001>-oriented island grains with different reductions: (**a**,**b**) 0%, (**c**,**d**) 20%, (**e**,**f**) 40%, (**g**,**h**) 60%, (**i**,**j**) 80%.

Figure 5 shows the microstructure of the XZ center section of pancake-like and spherical {210}<001> island grains under different reductions. At a reduction of 20%, the upper and lower layers of pancake-like {210}<001> island grains are more stable, and the overall deformation of spherical {210}<001> island grains is relatively uniform, while the matrix Goss orientations (green) rotate towards the {111}<112> orientations (blue) around the TD axis, as shown in Figure 5c,d. From Figure 5e, it can be observed that at 40% reduction, a “zigzag-shaped” deformation region appears within the pancake-like island grain, primarily consisting of the {114}<uvw> orientation (pink). However, the spherical {210}<001> island grain regions near the grain boundaries along the X direction are more stable than that within. At 60% reduction, grain boundaries are inclined, as shown in Figure 5g, where the upper and lower parts of the pancake-like {210}<001> island grain are distributed in an anti-symmetrical manner, with the surface layer forming the {111}<112> orientations. Compared with the pancake-like {210}<001> island grain, the spherical {210}<001> island grain exhibits less uneven deformation, and the ends of the grain in the XZ central section rotate from {113}<361> orientations to {114}<481> orientations, as displayed by Figure 6h. Under 80% reduction, as shown in Figure 5i, the sides of the pancake-like island grain rotate from {113}<361> orientations to {111}<112> orientations. However, the internal regions of the spherical island grains rotate towards the {111}<112> orientations, with only the remaining purple {114}<481> orientations near the grain boundaries.

Figure 6 shows the orientations of the pancake-like and spherical {210}<001> island grains under different reductions. To clearly express the pole figures’ information, the scatter points are enlarged and highlighted in Figure 6a,b,d. Before 60% reduction, the rotational patterns of orientations for island grans are essentially identical. Due to the smaller volume fraction of {210}<001> orientations in spherical island grains, there are fewer scattered points in the (001) pole figures. At a reduction of 60%, some 0–20° cube orientations are formed in both kinds of {210}<001> island grains, as shown in Figure 6g,h, but symmetric 0–20° cube orientations are formed only in the pancake-like {210}<001> island grain. At an 80% reduction, partial 0–30° cube orientations and orientations are observed in the spherical {210}<001> island grain shown in Figure 2j. However, as shown in Figure 2i, some 0–30° cube orientations and {111}<112> orientations, along with their symmetrical orientations, are formed in the pancake-like {210}<001> island grain.

### 3.2. CPFEM Microstructure and Orientations of {110}<112> Island Grain

**Figure 7:**
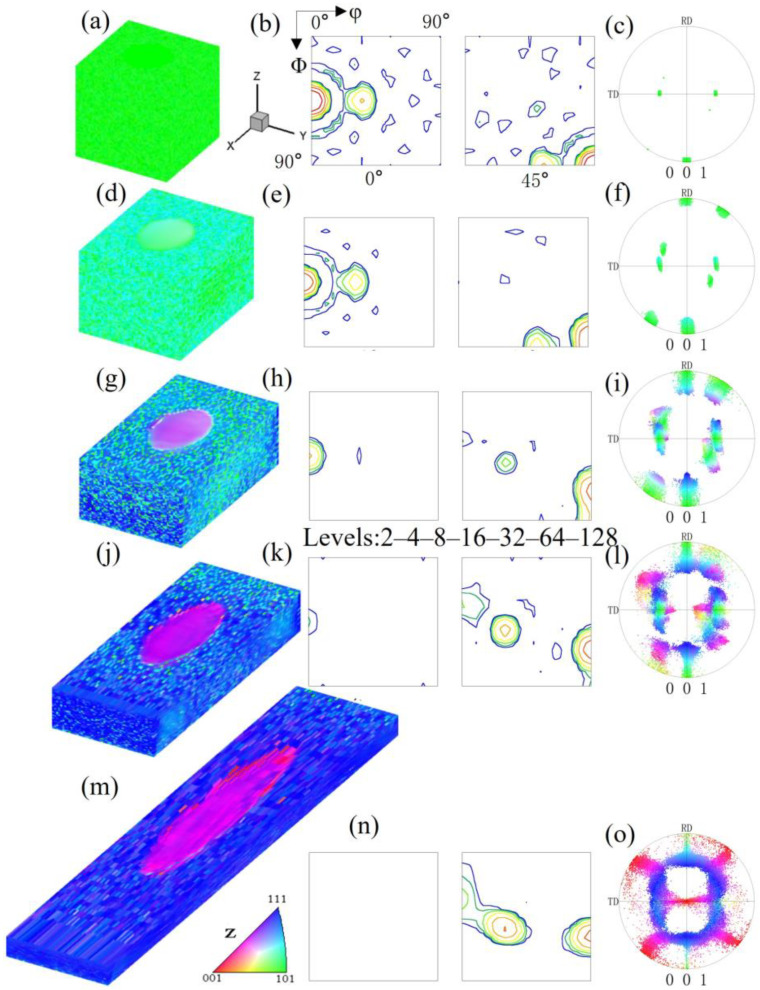
IPF-Z maps, ODF section and (001) pole figures of pancake-like {110}<112>-oriented island grains embedded in the Goss matrix with different reductions: (**a**–**c**) 0%, (**d**–**f**) 20%, (**g**–**i**) 40%, (**j**–**l**) 60%, (**m**–**o**) 80%.

Figure 7 shows the microstructure and orientation changes of pancake-like {110}<112> (Brass) island grains with Goss-oriented grain as the matrix under different reductions. Figure 7a is a map of the IPF-ND, so the color difference between the two grains is not discernible. However, the pole figure and ODF figure clearly delineate the differences between them. After a 40% reduction (Figure 7g), the Goss-oriented matrix rotates towards two {111}<112> variant orientations, with some residual micro-regions of the Goss orientation remaining. The {110}<112> island grain rotates towards the {112}<110> orientation. At 60% reduction (Figure 7j), two symmetrical {112}<110> variant orientations appear within the {110}<112> island grain. Under an 80% reduction, the island grain’s microstructure becomes relatively uniform, and the original Goss-oriented regions within the matrix gradually disappear, as shown in Figure 7e. The majority of the {110}<112>-oriented island grain regions rotate towards the α-fiber and gradually approach the {111}<112> orientation. In summary, the two island grain variant orientations are stable. Due to the slightly higher symmetry of the {110}<112> orientation, two {112}<110> variants are formed after plane strain compression.

**Figure 8:**
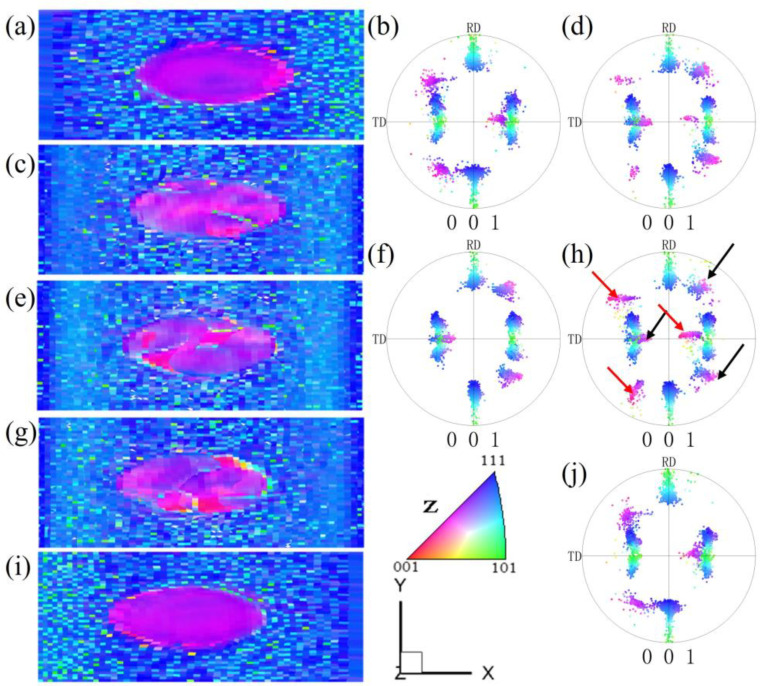
IPF-Z maps and (001) pole figures of XY section with {110}<112> island grains at 60% reduction: (**a**,**b**) z = 0, (**c**,**d**) z = 0.25, (**e**,**f**) z = 0.5, (**g**,**h**) z = 0.75, (**i**,**j**) z = 1.

Figure 8 shows the microstructure and orientations of the pancake-like {110}<112> island grain embedded in a Goss grain at 60% reduction. The Goss orientations rotate towards the {111}<112> orientations, with only a small amount residue. The internal inhomogeneity of the pancake-like {110}<112> island grain varies across different sections. In the upper and lower surfaces regions viewed from the XY cross-section, as shown in Figure 8a,i, most of the pancake-like {110}<112> island grain orientations rotate towards the same variant of the purple {112}<110> orientation. In the central layer shown in Figure 8e, the internal inhomogeneity of the island grains is higher, mainly rotating towards another variant of the {112}<110> orientation. In the z = 0.25 and z = 0.75 layers of the XY cross-section, the {110}<112> island grains also produce two variants of the {112}<110> orientations, as shown by the arrows in Figure 8h, where one color arrow represents one variant. There are also partial yellow {210}<001> orientations in the z = 0.75 layers of the XY cross-section.

**Figure 9:**
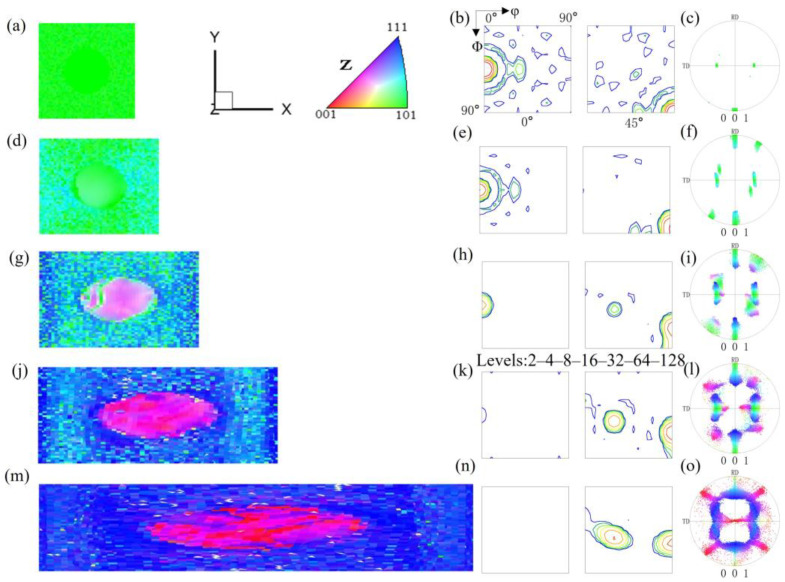
IPF-Z maps of the XY center section, ODF section and (001) pole figures of the spherical {110}<112>-oriented island grains embedded in the Goss matrix with different reductions: (**a**–**c**) 0%, (**d**–**f**) 20%, (**g**–**i**) 40%, (**j**–**l**) 60%, (**m**–**o**) 80%.

Figure 9 shows the central layer microstructure and overall orientation changes of spherical {110}<112> island grains within the Goss matrix under different reductions. Compared with the pancake-like island grain simulation block, the orientation rotation path is consistent, and the α-fiber texture ultimately generated by the spherical island grain simulation block is weaker. From the perspective of the center layer of the XY cross-section, the deformation inhomogeneity within the island grain is evident. The rotated cube’s orientation is distributed both within the island grain and near the grain boundaries. In the pancake-like island grain simulation block, a small amount of yellow {210}<001> orientation is formed in the center layer of the XY cross-section, without further rotation towards the rotated cube orientation around the ND axis.

**Figure 10:**
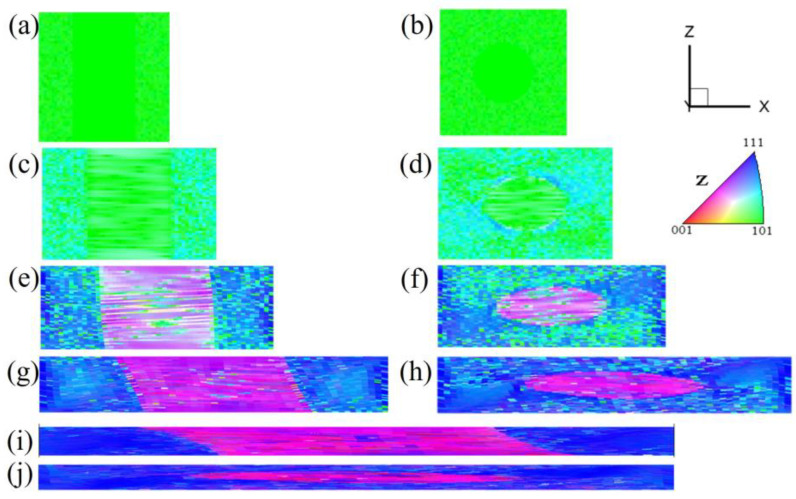
IPF-Z maps of XZ center section of pancake-like and spherical {110}<112> island grains with different reductions: (**a**,**b**) 0%, (**c**,**d**) 20%, (**e**,**f**) 40%, (**g**,**h**) 60%, (**i**,**j**) 80%.

Figure 10 shows the microstructure of the XZ center section of pancake-like and spherical {110}<112> island grains under different reductions. At a reduction of 20%, there is a significant difference in the deformation inhomogeneity of the Goss matrices around the {210}<001> island grains. The overall deformation of the pancake-like {210}<001> island grain is relatively uniform, but the Goss matrix around the spherical {210}<001> island grains rotates towards {111}<112> orientations. Compared with Figure 10e,f, it is found that the island grain orientation changes are consistent, and there is an obvious layering phenomenon in the lateral center layer of {110}<112> island grains. However, the stratification phenomenon of pancake-like {110}<112> island grains is still obvious before the 80% reduction is reached, but the spherical island grains do not have this feature.

**Figure 11:**
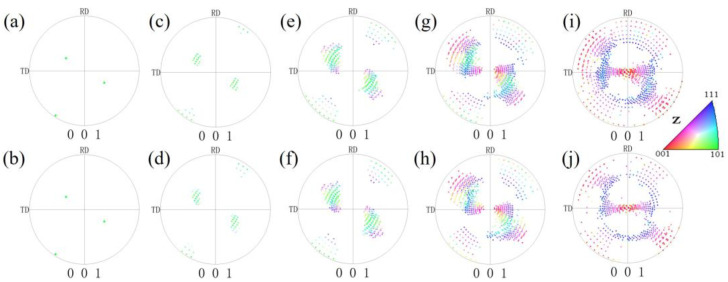
(001) pole figures of pancake-like and spherical {110}<112> island grains with different reductions: (**a**,**b**) 0%, (**c**,**d**) 20%, (**e**,**f**) 40%, (**g**,**h**) 60%, (**i**,**j**) 80%.

Figure 11 shows the orientations of the pancake-like and spherical {110}<112> island grains under different reductions. It is also found that the rotational patterns of orientations for island grans are essentially identical. The {110}<112> orientations rotate towards {111}<112> orientations and rotated cube orientations at 80% reduction. In addition, some 0–20° cube orientations are formed in the pancake-like {110}<112> island grains.

When comparing the microstructure and orientation changes between the pancake-like and spherical states of {210}<001>- and {110}<112>-oriented island grains, it is observed that the orientation scatters of the spherical island grains are reduced. This is likely due to the fact that there are more orientations within the spherical island grains that interact less with the surrounding Goss matrix. As a result, at the same reduction, the pancake-like island grains exhibit greater deformation inhomogeneity in the center layer of the XY cross-section.

### 3.3. CPFEM Microstructure and Orientations of {114}<481> and {100}<021> Island Grains

**Figure 12:**
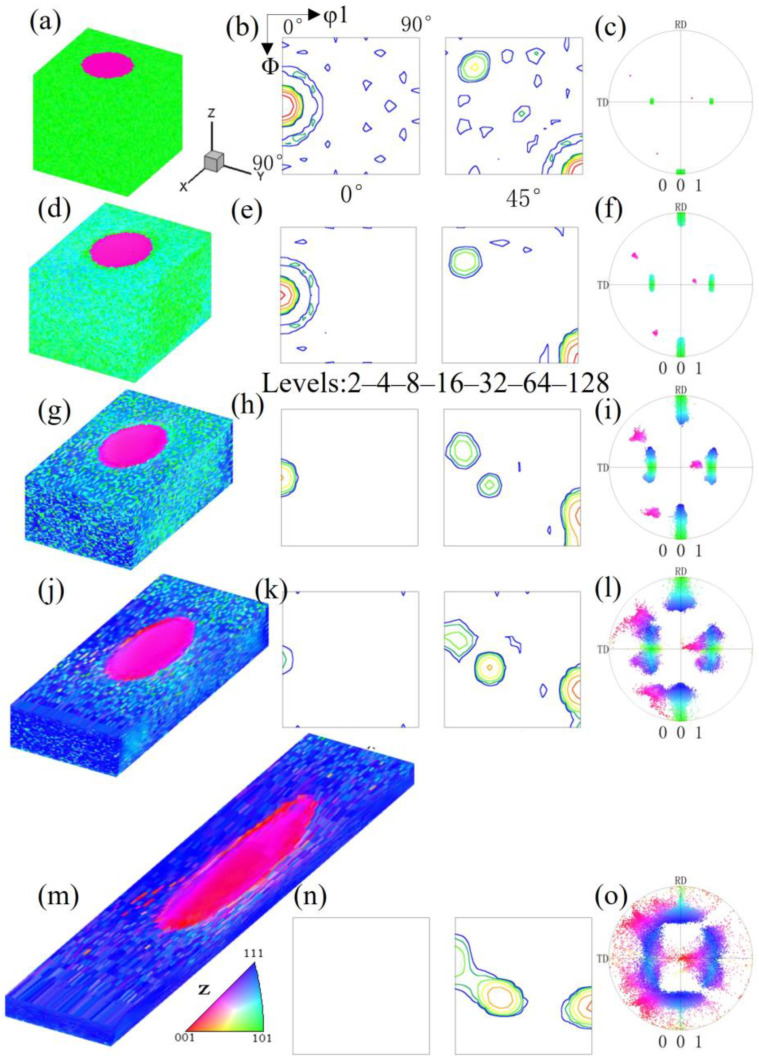
IPF-Z maps, ODF section and (001) pole figures of {114}<481> island grains embedded in the Goss matrix with different reductions: (**a**–**c**) 0%, (**d**–**f**) 20%, (**g**–**i**) 40%, (**j**–**l**) 60%, (**m**–**o**) 80%.

Figure 12 shows the orientation changes of a {114}<481> island grain within Goss matrix under plane strain compression. Figure 12a shows the initial microstructure and orientation. After a 20% reduction (Figure 12d), the orientations are relatively stable. As shown in Figure 12g, the Goss matrix clearly rotates towards two {111}<112> variant orientations under 40% reduction, with the residual Goss-oriented regions being unevenly distributed. The {114}<481> island grain slowly rotates towards the α-fiber. Under 60% reduction, as shown in Figure 12j, the residual Goss regions in the matrix are further reduced, and the island grain orientation reaches the basically stable rolling orientation of {112}<110>, but there is an orientation gradient within the island grain. It can be observed from Figure 12j–l that in the surface layer of the simulated block, the island grains form a small amount of near-35° rotated cube orientations. As depicted in the Figure 12m, there are still very few residual Goss regions under 80% reduction.

**Figure 13:**
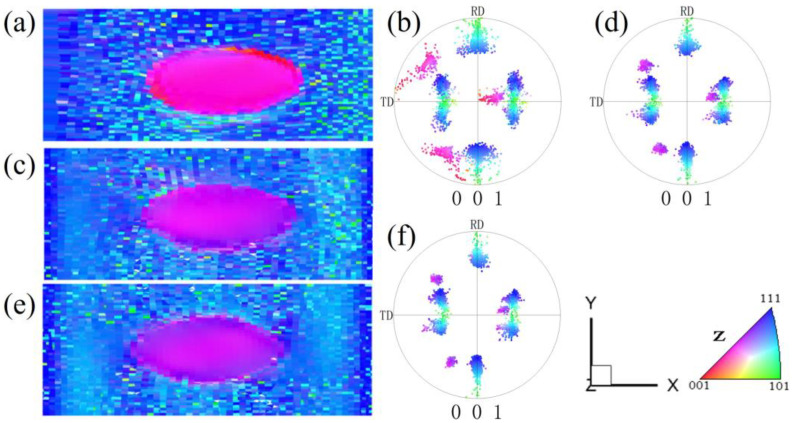
IPF-Z maps and (001) pole figures of XY section with {114}<481> island grains at 60% reduction: (**a**,**b**) z = 0, (**c**,**d**) z = 0.25, (**e**,**f**) z = 0.5.

Figure 13 shows the microstructure and orientation changes of {114}<481> island grains with Goss grains as the matrix at 60% reduction. The rotation pattern of the matrix Goss orientation is similar to that of the Goss orientation with other island grains. In the surfaces of the XY cross-section, as shown in Figure 13a, the {114}<481> island grain rotates towards the {113}<361> orientation, with a small amount of red near the 10° rotated cube orientation. In the z = 0.25 layer and central layer of the XY cross-section, as shown in Figure 13c,e, the {114}<481> island grains are relatively stable.

**Figure 14:**
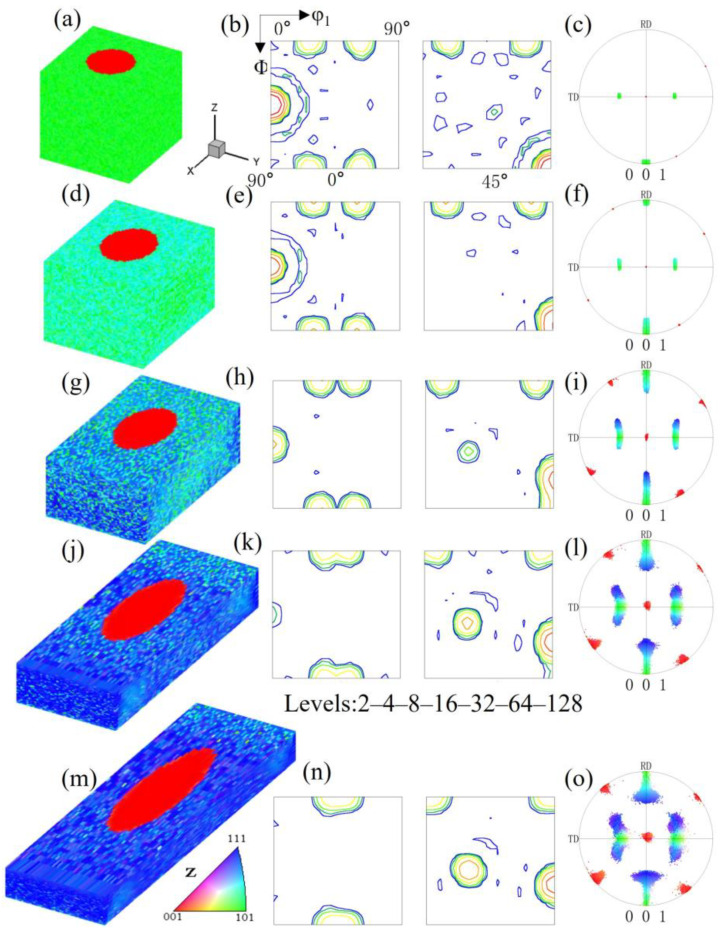
IPF-Z maps, ODF section and (001) pole figures of {100}<021> island grains embedded in the Goss matrix with different reductions: (**a**–**c**) 0%, (**d**–**f**) 20%, (**g**–**i**) 40%, (**j**–**l**) 60%, (**m**–**o**) 70%.

**Figure 15:**
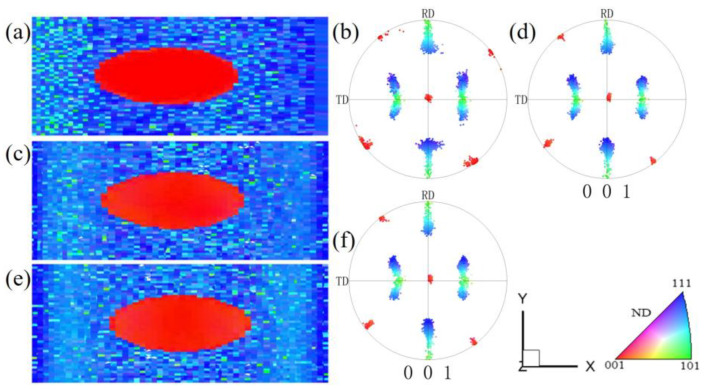
IPF-Z maps and (001) pole figures of XY section with {100}<021>-oriented island grains at 60% reduction: (**a**,**b**) z = 0, (**c**,**d**) z = 0.25, (**e**,**f**) z = 0.5.

Figure 14 shows the orientation changes of a {100}<021> island grains within the Goss matrix under plane strain compression. In Figure 14d, we see that the Goss orientations rotate around the TD axis towards the {111}<112> orientations at 20% reduction, while the {100}<021> island grain rotates around the ND axis. The orientation gradient within the island grain is significantly smaller than that of the Goss matrix. After 40% reduction, the matrix noticeably rotates towards two {111}<112> variants, with the residual Goss-oriented regions being evenly distributed, as shown in Figure 14g. The island grain orientation, with no obvious orientation gradient, continues to move towards the rotated cube orientation. Figure 14j shows that the residual Goss regions within the Goss matrix are further reduced under 40% reduction. Due to data divergence at 80% reduction, only the data after 70% reduction are provided in Figure 14m. In addition to the formation of two strong {111}<112> variants within the Goss matrix, there are very few residual Goss-oriented regions. The island grain rotates towards a near-rotated cube orientation, and thus, a significant deformation inhomogeneity region does not appear internally, thus failing to effectively promote the nucleation of recrystallization during subsequent annealing. No inhomogeneous deformation region within the island grain is observed through the XY cross-sectional microstructure and orientation changes at 60% in Figure 15.

## 4. Discussion

We have previously conducted a comparison between experimental measurements and simulations regarding the orientation rotation pattern of Goss grains during plane strain compression [40], indicating that Goss grains tend to rotate towards two complementary {111}<112> variants, with the residual Goss regions located between the two variants, predominantly distributed in a band-like manner. This paper also examines whether the rotations of the four different orientation island grains affect the rotations of the adjacent Goss grains.

### 4.1. Comparison of Simulation Results with Experimental Results

**Figure 16:**
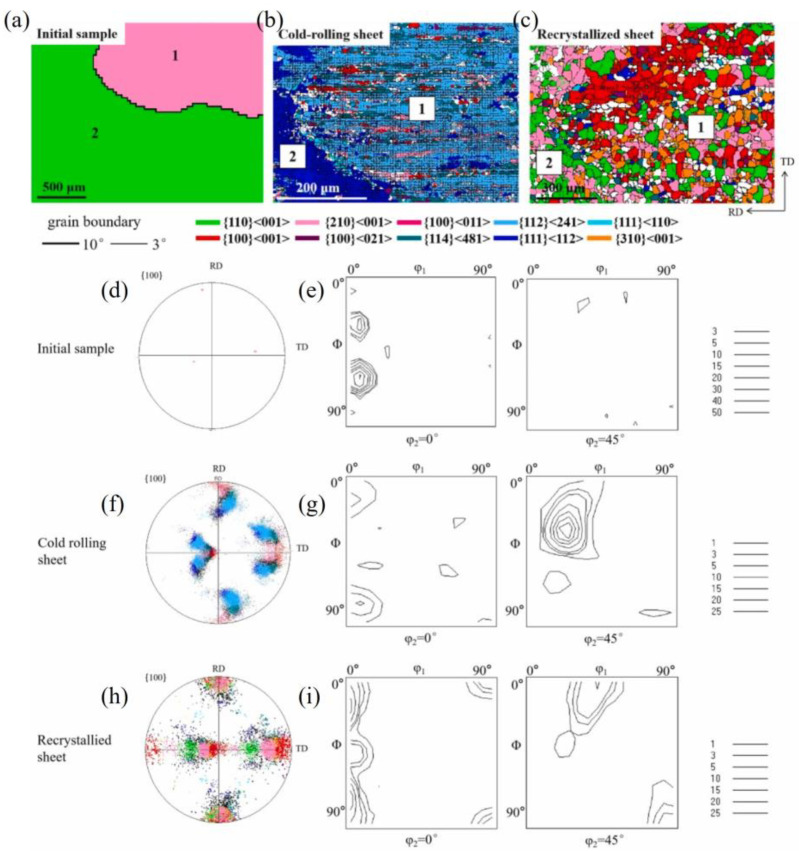
Evolution of {210}<001> island grains during cold rolling at 70% and annealing (930 °C, 3 min): (**a**,**d**,**e**) the original EBSD data of the island grain, (**b**,**f**,**g**) EBSD data after cold rolling, (**c**,**h**,**i**) EBSD data after recrystallization. Reproduced with permission from Ping Yang, Materials Chemistry and Physics; published by Elsevier, 2022 Reference [12].

Figure 16 is an example of a nearly {210}<001> island grain surrounded by Goss grains after cold rolling and recrystallization, as we previously measured [12]. After being cold-rolled to 70%, the {210}<001> island grain was seen to rotate mainly towards the {112}<241> orientation, while the surrounding Goss matrix rotated towards the {111}<112> orientations. As seen in Figure 16a, there are also small amounts of cube (red), {114}<481> (dark green), {210}<001> (pink), and {111}<112> (dark blue) orientations. When observed from the rolling plane, there is a band-like structure parallel to the RD, which represents the transition bands of various orientations. After a 60% plane strain compression simulation of the {210}<001> island grain, the XY section also exhibits band-like regions parallel to the RD, mainly including the {210}<001> orientation and near-{111}//ND orientations (Figure 3). After recrystallization annealing, the {210}<001> island grains are predominantly characterized by cube, {310}-{210}<001>, and Goss grains, as seen in Figure 16c,h,i. That is to say, this island grain has developed favorable recrystallization orientations for ultra-thin strips. The cube orientation is likely the transitional orientation between the two deformed {112}<421> variants. Reference [2] also presents an example wherein the island grain almost entirely shows cube orientation. The reason for the formation of cube grain clusters may be that the island grain is greatly influenced by the interaction of the matrix. This leads to intense internal deformation within the island grains, with transition bands or regions of inhomogeneous deformation repeatedly forming. As a result, there are many transition bands of cube orientation, which ultimately leads to the formation of an entire island grain region consisting of cube orientation.

**Figure 17:**
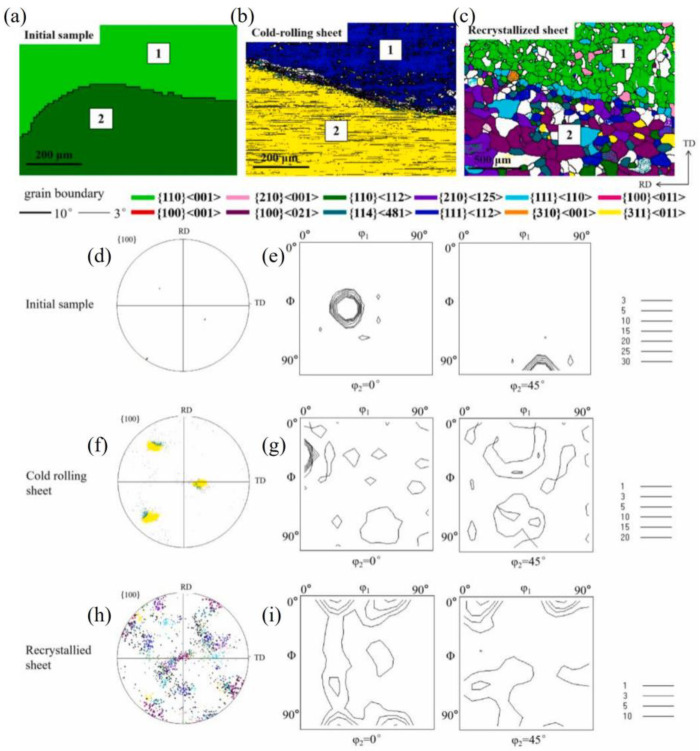
Evolution of {110}<112> island grains during cold rolling to 70% and annealing (930 °C, 3 min): (**a**,**d**,**e**) the original EBSD data of island grain, (**b**,**f**,**g**) EBSD data after cold rolling, (**c**,**h**,**i**) EBSD data after recrystallization. Reproduced with permission from Ping Yang, Materials Chemistry and Physics; published by Elsevier, 2022 in Reference [12].

Figure 17 is an example of the orientation changes of the {110}<112> island grain surrounded by Goss grains after cold rolling and recrystallization, which we previously measured [12]. It can be observed that after 70% cold rolling, the {110}<112> island grain rotates towards the {113}<110> orientation, but there is no significant presence of the {111}<110> orientation, as seen in Figure 17b,f,g. It is possible that the 70% reduction is not high enough. There is a lack of internal orientation gradient, and the deformation is relatively uniform. The surrounding Goss matrix rotates towards the {111}<112> orientations. After recrystallization annealing, the main orientations formed are {100}<021> (purple) and {120}<215> orientations, with a small amount of {110}<112> orientation and {111}<110> orientation, as seen in Figure 17c,h,i. The grain size in Figure 17c is relatively large, and the texture intensity is low, indicating that there should be a small number of micro-regions with complex orientations, leading to the formation of a coarser grain at a low nucleation rate. In addition, the main texture {100}<021> is also a favorable recrystallization texture. Compared with the simulated microstructure in Figure 4, the {113}<110> texture is consistent. The microstructure observed from the rolling plane in the experiment is more uniform, while the simulation provides a side view (Figure 11), showing more horizontal band structures. Compared with the rolling test of coarse {110}<112>-oriented grains [47], the main textures after recrystallization are weak {111}<112> and {112}<110> textures.

At present, there are no measured data on the deformation of {114}<481> and {100}<021> island grains within Goss-oriented grains, making it difficult to compare simulated data with them. For high-grade non-oriented silicon steel normalized sheets, the main texture is {114}<481> [48,49]. After cold rolling, it rotates to the α-fiber. After recrystallization, it returns to the α*-fiber, with {114}<481> and {100}<021> textures as the main ones. This is similar to the island grain deformation simulation in this paper, but these two orientations are rare in the deformed microstructure.

Our previous research investigated the orientation changes that occur during rolling, and the characteristics of recrystallization in the deformed inhomogeneous regions after the annealing of {100}<021>-oriented grains within {100} columnar grains [50]. The results show that {100}<021>-oriented grains gradually rotated towards {114}<481> or {113}<361> orientations during rolling. With further increases in rolling reductions, the α*-fiber orientations will rotate to the {112}<110> orientation [50]. Additionally, it was also observed in Reference [50] that the initially adjacent {100}<011>- and {100}<021>-oriented columnar grains were rolled to 90% reduction, and annealed for a short time. The former had a stable orientation and uniform microstructure, while the latter moved to stable deformation {112}<110> orientation, and new grains with various orientations such as {113}<361> and {100}<021> were formed at the grain boundaries with {100}<011> deformed grains. This is different from the fact that the {100}<021> island grains in this paper rotated towards the rotated cube orientation, as shown in Figure 14 and Figure 15. One reason for this difference is presumed to be the low level of interaction between grains in the simulation, with 70% reduction. This is also reflected in the plane strain compression simulation of {100} columnar grains. The second reason is that the selected {100}<021> orientation is an ideal orientation with high symmetry, while the actual measured {100}<021> grains have some orientation deviations, making the orientation less stable and more prone to deviation from the <100>//ND orientations during rolling. Further research is needed to determine whether there is a significant difference in deformation between island grains with these orientations and coarse columnar grains.

**Figure 18:**
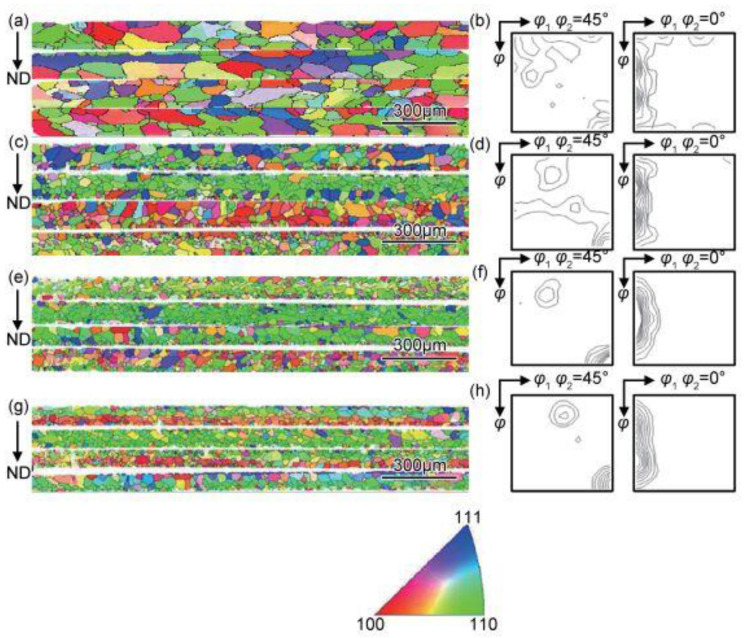
IPF-Z maps and ODF section of samples with lower initial magnetic inductions after annealing at 850 °C for 5 min at different reductions: (**a**,**b**) 60%, (**c**,**d**) 65%, (**e**,**f**) 70%, (**g**,**h**) 75%. Reproduced with permission from Ping Yang, Journal of Materials Engineering; published by Journal of Materials Engineering Editorial Department, 2017 [8].

Figure 18 shows the EBSD data of the conventional grain-oriented silicon steel finished sheets with low initial magnetic properties from Reference [8] after annealing at 850 °C for 5 min. It can be observed that, after annealing following 60% reduction, there are some large-sized recrystallized grains close to the <100>//ND and α*-fiber orientations, which are presumably inherited from the residual near-<100>//ND- and near-{114}<481>-oriented regions after rolling. As the rolling reductions increase, the recrystallized grains become finer, indicating an increase in nucleation rate. The grains close to the <100>//ND and near-{114}<481> orientations are distributed in chains, similar to the scenario in Figure 15, with more data found in Reference [12]. The presence of these clusters of non-Goss-oriented grains may replace the Goss-oriented grains during subsequent high-temperature annealing [8]. The stronger the Goss texture after primary recrystallization, the easier it is for other weak texture components to replace the Goss texture as the main secondary recrystallization texture during subsequent high-temperature annealing [6]. If the weak texture component is the cube texture, then we obtain the desired texture. This is one of the significant findings of the research in this paper, although further experimental verification is needed. In Figure 18, the main textures are the Goss texture and the {210}<001> texture, while the secondary texture is the near-{114}<481> texture. The Goss texture is inherited from the deformation and recrystallization of the original accurate Goss grains, while the {210}<001> texture could be inherited from the deformation of the large grains sheets with a deviated Goss orientation, or it could be produced by the deformation and recrystallization of island grains of this orientation. The near-{114}<481> orientation should be associated with the initial grains of similar orientations and <100>//ND orientations.

### 4.2. Possible Application of Cube Grain Clusters Due to Island Grains

Island grains in grain-oriented silicon steel finished sheets have a detrimental effect on magnetic properties due to their different orientations from Goss orientations, and there has been considerable research on their origins [2,3]. However, their deformation and recrystallization behaviors in ultra-thin strips have received little attention. Most studies on grain-oriented steel ultra-thin strips focus on the sharpness of the initial Goss texture or the impact of grain size on magnetic properties after cold rolling and annealing [6,51]. Depending on the final annealing temperature of the ultra-thin strips, secondary recrystallization or even tertiary recrystallization microstructures may occur [52], and the primary recrystallization microstructure and texture will significantly influence the secondary recrystallization microstructure and texture. Not only do the initially coarse, deviated Goss grains behave differently after cold rolling and annealing compared to the accurate Goss grains, but island grains also exert a different influence due to their special orientation and size. Among the four types of island grains selected in this study, two are secondary grain orientations, namely, {210}<001> and {110}<112>; the other two are primary recrystallization grain orientations, namely, {114}<481> and {100}<021>, and the impacts they produce are different. Although these four types of island grains can all produce <100>//ND-oriented grains, their sharpness varies. Compared to the recrystallized Goss grains, they do not enhance magnetic properties, but can affect the secondary recrystallization texture. Special attention should be paid to which type of island grain produces a strong cube grain cluster. From the deformed microstructure inspection, it can be seen that the initial {210}<001> island grains have the highest probability of producing such cube grain clusters. They are the most susceptible to forming cube-oriented transition bands under the influence of grain boundaries, and have strong recrystallization nucleation capabilities, thus they form the sharpest cube-oriented grain clusters. If a large number of such island grains exist, the magnetic properties may be improved after recrystallization. This is because strong cube grains have two <100> directions on the sheet’s surface, which can reduce the magnetic anisotropy in different directions, superior to the Goss texture with only one <100> direction. As for whether strong cube texture sheets can be obtained through further annealing or secondary recrystallization from these cube grain clusters, further research is needed.

## 5. Conclusions

Using four types of island grains with particular orientations of {210}<001>, {110}<112>, {114}<481>, and {100}<021>, which were modeled within the Goss-oriented matrix using the full-field CPFEM simulation under plane strain compression, the orientation evolution and deformation heterogeneity of island grains and Goss matrix grains are analyzed quantitatively and compared with corresponding experimental results. This study considers cold rolling as uniform rolling, approximates it with plane strain compression, and does not take into account the friction between the rolls and the surfaces of the sheet.

(1) The four orientations of island grains show different orientation changes and inhomogeneous deformation regions under plane strain compression. The fragmentation of the predominant {210}<001> island grain generates shear bands at a certain angle to the rolling direction, which is very similar to the actual measured data, with a small number of {100}<021> orientations. The rotation speed of the {110}<112> island grain is lower than that of the {210}<001> island grain. Its inhomogeneous deformation regions are characterized by a horizontal layered distribution, which is distinctly different from the other orientation island grains. A clear orientation gradient along the ND is observed, correlating well with the actual grain deformation orientation. The {114}<481> and {100}<021> island grains exhibit slow orientation rotation speeds, and their internal deformations are highly uniform, making it difficult to form significantly inhomogeneous deformation regions. It is speculated that the promotion effect on subsequent recrystallization nucleation is relatively small, providing basic evidence for the industrial control of {114}<481> and {100}<021> island grains.

(2) Comparative analysis between the pancake-like and spherical {210}<001> and {110}<112> island grains shows that the pancake-like cylindrical island grains have a higher degree of orientation dispersion compared to the spherical island grains. Furthermore, the deformation inhomogeneity within the center layer of the XY section of the pancake-like island grains is also more pronounced. It was determined that the deformation of island grains is different from that of grains with the same orientation in ordinary steel sheets.

(3) In the full-field CPFFEM results, the initial {210}<001> island grains are particularly prone to forming a cube orientation transition region due to grain boundary effects. Combining the in-situ experimental outcome, the cube orientation transition may form the sharpest cube-oriented grain clusters, which has theoretical significance for improving the magnetic anisotropy of silicon steels. Therefore, when selecting the base material containing island grains for preparing ultra-thin strips, oriented silicon steels containing {210}<001> island grains should be chosen.

## Figures and Tables

**Table 1:** The CPFEM parameters for the {100} columnar grains’ microstructure [42,45,46].

C_11_ (GPa)	C_12_ (GPa)	C_44_ (GPa)	τ_0_ (MPa)	Τ_s_ (MPa)	m	H_0_ (MPa)	${\dot{\gamma }}_{0}\;(s^{-1})$	${\dot{\gamma }}_{s}\;(s^{-1})$	n
232.2	135.6	117.0	22.2	150.2	0.075	553	0.01	5 × 10^10^	0.05

## Data Availability

The data that support the findings of this study are available from the corresponding author upon reasonable request.
